# The relations between different components of intolerance of uncertainty and symptoms of depression during the COVID-19 pandemic: A network analysis

**DOI:** 10.3389/fpsyt.2022.993814

**Published:** 2022-10-13

**Authors:** Tingwei Feng, Lei Ren, Chang Liu, Kuiliang Li, Lin Wu, Xinyi Wei, Shangqing Yuan, Long-Biao Cui, Xi Yang, Danyang Li, Wei Yang, Ye Li, Buyao Wang, Hui Wang, Xufeng Liu

**Affiliations:** ^1^Military Medical Psychology School, Fourth Military Medical University, Xi'an, China; ^2^BrainPark, Turner Institute for Brain and Mental Health and School of Psychological Sciences, Monash University, Clayton, VIC, Australia; ^3^School of Psychology, Army Medical University, Chongqing, China; ^4^Department of Psychology, Renmin University of China, Beijing, China; ^5^School of Psychology, Capital Normal University, Beijing, China; ^6^Department of Health Economy Management, Xijing Hospital, Fourth Military Medical University, Xi'an, China; ^7^College of Education Science, Changji University, Changji, China; ^8^Psychological Counseling Center, Xijing University, Xi'an, China; ^9^Clinical and Psychological Counseling, DongFang College, Beijing University of Chinese Medicine, Langfang, China

**Keywords:** COVID-19, depression, intolerance of uncertainty, network analysis, central nodes, bridge nodes

## Abstract

**Background:**

The relations between depression and intolerance of uncertainty (IU) have been extensively investigated during the COVID-19 pandemic. However, there is a lack of understanding on how each component of IU may differentially affect depression symptoms and vice versa. The current study used a network approach to reveal the component-to-symptom interplay between IU and depression and identify intervention targets for depression during the COVID-19 pandemic.

**Methods:**

A total of 624 college students participated in the current study. An IU-Depression network was estimated using items from the 12-item Intolerance of Uncertainty Scale and the Patient Health Questionnaire-9. We examined the network structure, node centrality, and node bridge centrality to identify component-to-symptom pathways, central nodes, and bridge nodes within the IU-Depression network.

**Results:**

Several distinct pathways (e.g., “Frustration when facing uncertainty” and “Feelings of worthlessness”) emerged between IU and Depression. “Fatigue” and “Frustration when facing uncertainty” were identified as the central nodes in the estimated network. “Frustration when facing uncertainty,” “Psychomotor agitation/retardation,” and “Depressed or sad mood” were identified as bridging nodes between the IU and Depression communities.

**Conclusion:**

By delineating specific pathways between IU and depression and highlighting the influential role of “Frustration when facing uncertainty” in maintaining the IU-Depression co-occurrence, current findings may inform targeted prevention and interventions for depression during the COVID-19 pandemic.

## Introduction

The COVID-19 pandemic has developed into a global public health emergency (1). The pandemic has brought serious psychosocial stressors (e.g., lockdowns, keeping social distance, loss of livelihood, and decreases in economic activity), which could be dangerous for public mental health (2, 3). Specifically, these stressors may drive risks for the onset and development of depression symptoms (4). A recent meta-analysis found that depression was prevalent during the COVID-19 pandemic, with a prevalence rate of 33.7% (5). Owing to the COVID-19 pandemic, the global prevalence of the major depressive disorder has increased by about 27.6% (6). The COVID-19 pandemic has led to significant uncertainty for the general public (7). The detrimental effects of pandemic-related uncertainties may be particularly relevant to individuals with high levels of intolerance of uncertainty (IU), who are prone to present negative cognitions, emotions, and behaviors when facing unpredictable events (8, 9). This mental health inequality is well-documented in the literature, with IU being consistently identified as a predictor of depression severity during the COVID-19 pandemic (7, 10–12).

Despite the robust associations between IU and depression (9), there are limited insights into how specific components of IU are related to individual depression symptoms. Specifically, prior research tends to use the latent variable approach when estimating the relationships between IU and depression. The approach treated both IU and depression as unitary constructs (indexed by sum scores of IU instruments and depression instruments) and either compared differences in IU between depressed and non-depressed groups (based on the cut-off value of depression symptom sum scores) or examined IU in relation to depression severity (7, 9–12). Concerns have been raised over treating IU and depression as unitary constructs. Specifically, depression is a heterogeneous syndrome consisting of various symptoms (e.g., fatigue, sad mood, and appetite changes), which differ from each other in important domains [e.g., predisposing factors, (13, 14)]. Individual depression symptoms have shown different connections with insomnia (15), internet addictions (16), traumatic stress (17), abuse (18), negative life events (19), and emotion regulation difficulties (20, 21). Similarly, the heterogeneity of components that constitute IU has been observed in previous studies (22, 23). And individual IU components have shown different connections with different symptoms of anxiety (24) and problematic smartphone use (25). Hence, treating IU and depression as unitary constructs (using sum scores) may overlook their relationships at the component-to-symptom level, hindering conceptual understanding of mechanisms underlying the co-occurrence of IU and depression.

To address the aforementioned concerns, the current study adopted the network approach to explain the co-occurrence of IU and depression. From a network perspective, psychopathology may be viewed as a network consisting of interacting variables (nodes) and pathways (edges) among them (26, 27). Components of IU and symptoms of depression may directly interact with one another (*via* distinct symptom pathways) and result in the co-occurrence of IU and depression. By inspecting the network structure, researchers may delineate specific pathways through which constructs interact and reinforce each other. Further, network analysis provides novel indices to understand the role played by each node within the network (28). For instance, nodes with high “expected influence” are highly connected to the remaining nodes within the network, thus, may serve to maintain the network. Meanwhile, “bridge expected influence” quantifies nodes' cross-construct connectivity. Thus, nodes with high “bridge expected influence” are considered the key to the maintenance of co-occurrence (29).

To our knowledge, no study to date has examined how individual IU components may contribute to specific depression symptoms. To address this gap and extend previous research on the IU-Depression association, the current study modeled the component-to-symptom relationships between IU and depression *via* the network approach. In the present study, we incorporated different components of IU and symptoms of depression into one network. This study had three goals: (1) elucidate component-to-symptom pathways between IU and depression, (2) identify central nodes within the IU-Depression network, and (3) identify influential bridge nodes connecting IU and depression communities.

## Methods

### Study population and survey design

Due to the COVID-19 outbreak, we conducted this online survey between 16 and 18 December 2020 *via* Wenjuanxing (www.wjx.cn). A WeChat (one of the largest instant messaging applications in China) message with links to the online survey was sent to all participants. Permission was gathered before the survey even began. The study only accepted participants who gave their consent. In the present network, we need to estimate 21 nodes (i.e., 12 components of IU and nine symptoms of depression) and 210 possible edges (i.e., each node has a connection with all other nodes). Although there are no definite guidelines yet as to how many participants we need per parameter, a rule of thumb put forward was the number of participants needed typically exceeds the possible parameters (30). Thus, the present network may need to recruit at least 231 participants. A total of 633 university students from Xijing University participated in our study. All of these participants were Chinese-speaking undergraduate students. Nine questionnaires were excluded due to their demographic information being incomplete. Finally, 624 questionnaires in all were collected. The First Affiliated Hospital of the Fourth Military Medical University's Ethics Committee authorized both this study and the format of the online survey (Project No. BWS16J012). The final sample consisted of 624 participants [57% female, mean age = 19.38, standard deviation (SD) = 1.12, range = 18–25 years].

### Measures

#### Symptoms of depression

The Patient Health Questionnaire-9 (PHQ-9) is a self-assessment scale assessing depression symptoms over the past 2 weeks (31). This scale includes nine symptoms based on the diagnosis of DSM-IV depressive disorders and is widely used as a screening tool for clinical practice and research (31). Each item had responses ranging from 0 (not at all) to 3 (nearly every day). The PHQ-9 has been well-validated in Chinese college students (32). The scale showed good reliability in the current study (Cronbach's α = 0.89).

#### Components of IU

The 12-item Intolerance of Uncertainty Scale (IUS-12) is a short, efficient scale for assessing IU (33). This scale measures a variety of uncertainty-related beliefs, emotions, and behaviors, such as “Frustration when facing uncertainty” and “Smallest doubt can stop me from acting” (33). Responses to each item ranged from 1 (“not at all characteristic of me”) to 5 (“entirely characteristic of me”). In the present study, the Chinese version of IUS-12 was used to assess different components of IU (34). The Chinese version of IUS-12 has good reliability and validity. The scale used in the current study demonstrated good reliability (Cronbach's α = 0.84).

### Network analysis

The IU-Depression network was estimated using the Gaussian graphical model (GGM) (35). The GGM was estimated on the basis of non-parametric Spearman rho correlation matrices (36, 37). Within a GGM, the edge represents the partial correlation between nodes after controlling for all other nodes in the network (36). By using the graphical LASSO (Least Absolute Shrinkage and Selection Operator) algorithm, a regularized GGM was obtained (38). In this regularization process, trivially small correlations were shrunk to zero. This regularization approach may reduce “false positive” edges and result in a network that is more stable and interpretable (36, 38). At the same time, the hyperparameter was set to 0.5 to balance the trade-off between sensitivity and specificity (36, 39). The final network was constructed and visualized [Fruchterman-Reingold algorithm, (40)] by conducting the *R-package qgraph* (41).

To calculate the node expected influence for each node within the final network, the R-package *qgraph* was used (41). Node expected influence is the sum of the edge weights linking to a specific node (42). A node with a higher expected influence is considered statistically more important within the network. The R-package *networktools* were used to compute the node bridge expected influence for each node within the final network (29). Node bridge expected influence is the sum of the edge weights linking a specific node to all nodes within the opposite community. A node with a higher bridge expected influence may be more likely to activate the opposite community (29). There were two communities of nodes in the current network, namely, the IU community (12 items from the IUS-12) and the depression community (9 items from the PHQ-9).

We tested the precision and robustness of the final network using the R package *bootnet* (30). The accuracy of edge weights was examined *via* 2,000 bootstrap samples in a non-parametric bootstrap technique. The correlation stability (CS)-coefficient was used to quantify the stability of node centralities (i.e., node expected to influence and bridge expected influence). Using 2,000 bootstrap samples, a case-dropping bootstrap methodology was used to get the CS coefficients for both metrics. The recommended value for CS-coefficient is above 0.5 and should not be lower than 0.25 (30). We also conducted bootstrapped difference tests to examine the difference between two edge weights or two node centralities.

## Results

### Descriptive statistics

The common age of the 624 college students (57% female) is 19.38 ± 1.12 years (mean ± SD, varying from 18 to 25 years). Moreover, 246 individuals are sole offspring and 378 individuals are non-sole offspring. The mean scores on the IUS-12 and PHQ-9 are 35.08 ± 7.44 (mean ± SD, range from 15 to 55) and 6.04 ± 4.74 (mean ± SD, range 0–27), respectively. Table 1 listed each variable's abbreviation, mean scores, and standard deviations.

**Table 1:** Abbreviations, mean scores, and standard deviations for each variable selected in the present network.

**Variables**	**Abbreviation**	***M***	**SD**
**Components of intolerance of uncertainty**			
IUS-12-1: Unforeseen events upset me greatly	IU1	2.97	1.05
IUS-12-2: It frustrates me not having all the information I need	IU2	2.93	1.06
IUS-12-3: One should always look ahead so as to avoid surprises	IU3	3.52	0.94
IUS-12-4: A small, unforeseen event can spoil everything, even with the best of planning	IU4	2.90	1.01
IUS-12-5: I always want to know what the future has in store for me	IU5	3.23	1.09
IUS-12-6: I can't stand being taken by surprise	IU6	2.81	1.01
IUS-12-7: I should be able to organize everything in advance	IU7	3.35	0.95
IUS-12-8: Uncertainty keeps me from living a full life	IU8	2.67	1.06
IUS-12-9: When it's time to act, uncertainty paralyzes me	IU9	2.80	1.08
IUS-12-10: When I am uncertain I can't function very well	IU10	2.88	1.07
IUS-12-11: The smallest doubt can stop me from acting	IU11	2.58	1.07
IUS-12-12: I must get away from all uncertain situations	IU12	2.44	0.99
**Symptoms of depression**			
PHQ-1: Anhedonia	D1	0.78	0.69
PHQ-2: Depressed or sad mood	D2	0.73	0.69
PHQ-3: Sleep difficulties	D3	0.81	0.84
PHQ-4: Fatigue	D4	0.89	0.76
PHQ-5: Appetite changes	D5	0.71	0.84
PHQ-6: Feeling of worthlessness	D6	0.66	0.73
PHQ-7: Concentration difficulties	D7	0.81	0.73
PHQ-8: Psychomotor agitation/retardation	D8	0.45	0.66
PHQ-9: Thoughts of death	D9	0.20	0.49

M, mean; SD, standard deviation.

### Network structure

**Figure 1:**
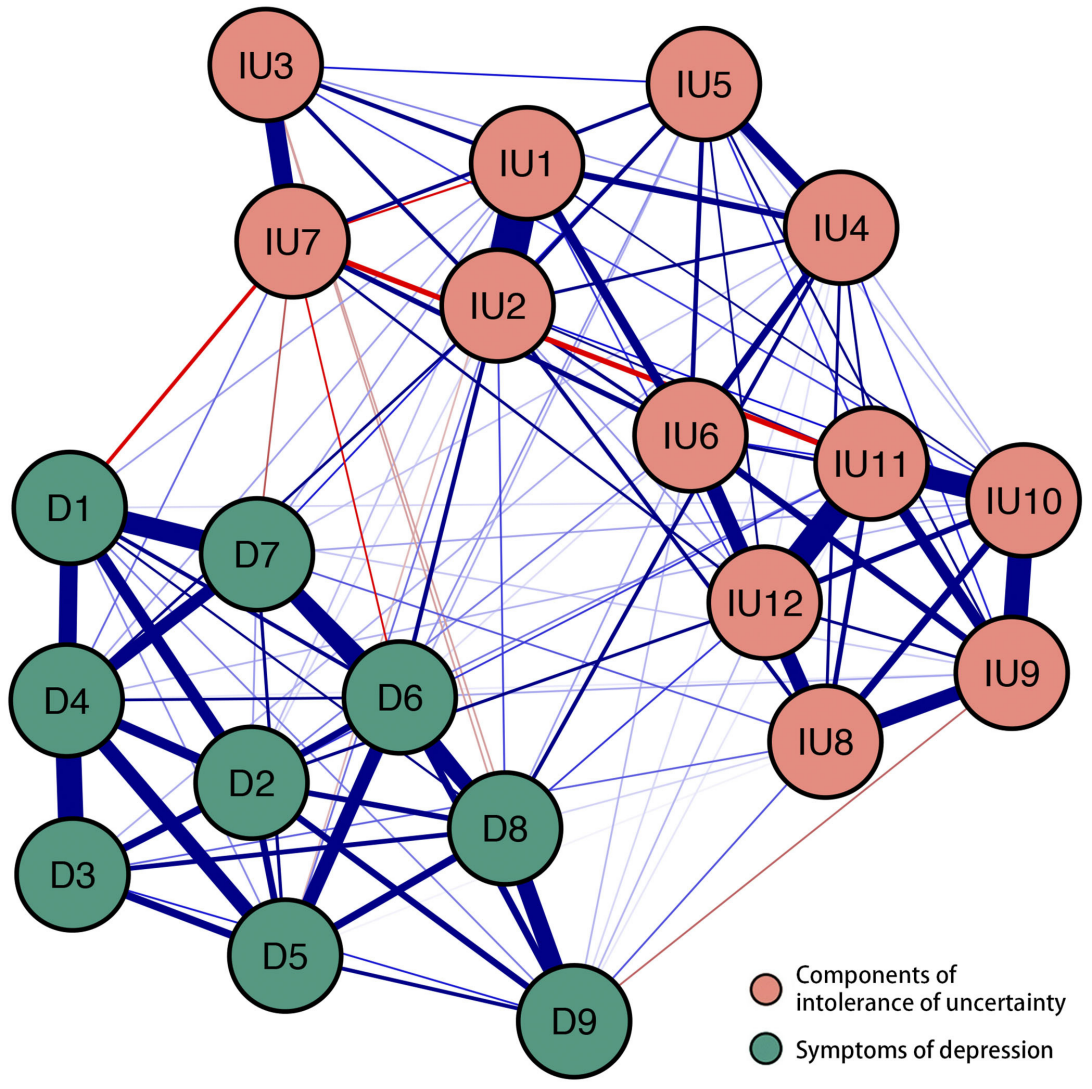
The network structure of different components of intolerance of uncertainty and symptoms of depression. Positive correlations were shown by blue borders, whereas negative correlations were represented by red edges. The size of the correlation was reflected in the thickness of the edge. Cut value = 0.05. The text of intolerance of uncertainty and depression can be seen in Table 1.

The final network was shown in Figure 1. There were several characteristics of this network. First, 127 edges were not zero (about 60%) among 210 possible edges and most of these edges were positive. And we found the six strongest edges in the final network. Among these six strongest edges, four edges were between IU's components IU1 and IU2 (weight = 0.40), IU11 and IU12 (weight = 0.30), IU9 and IU10 (weight = 0.27), IU10 and IU11 (weight = 0.26), and two edges were between D3 and D4 (weight = 0.28), D1 and D7 (weight = 0.24). It is worth noting that these six strongest edges had no one who connects IU's components and depression symptoms. Second, in the 108 possible edges between components of IU and symptoms of depression, 45 edges were not zero that ranged from −0.07 to 0.07. Four strongest edges were between IU2 and D6 (weight = 0.07), IU4 and D8 (weight = 0.07), IU12 and D2 (weight = 0.06), and IU2 and D4 (weight = 0.06). The two weakest edges were between IU7 and D1 (weight = −0.07) and IU7 and D6 (weight = −0.05). Supplementary material 2 showed the values of regularized partial correlation of all edges in the network. Supplementary Figure 1 showed the bootstrapped 95% confidence interval of edge weights and Supplementary Figure 2 showed the bootstrapped difference test for edge weights.

### Node expected influence

**Figure 2:**
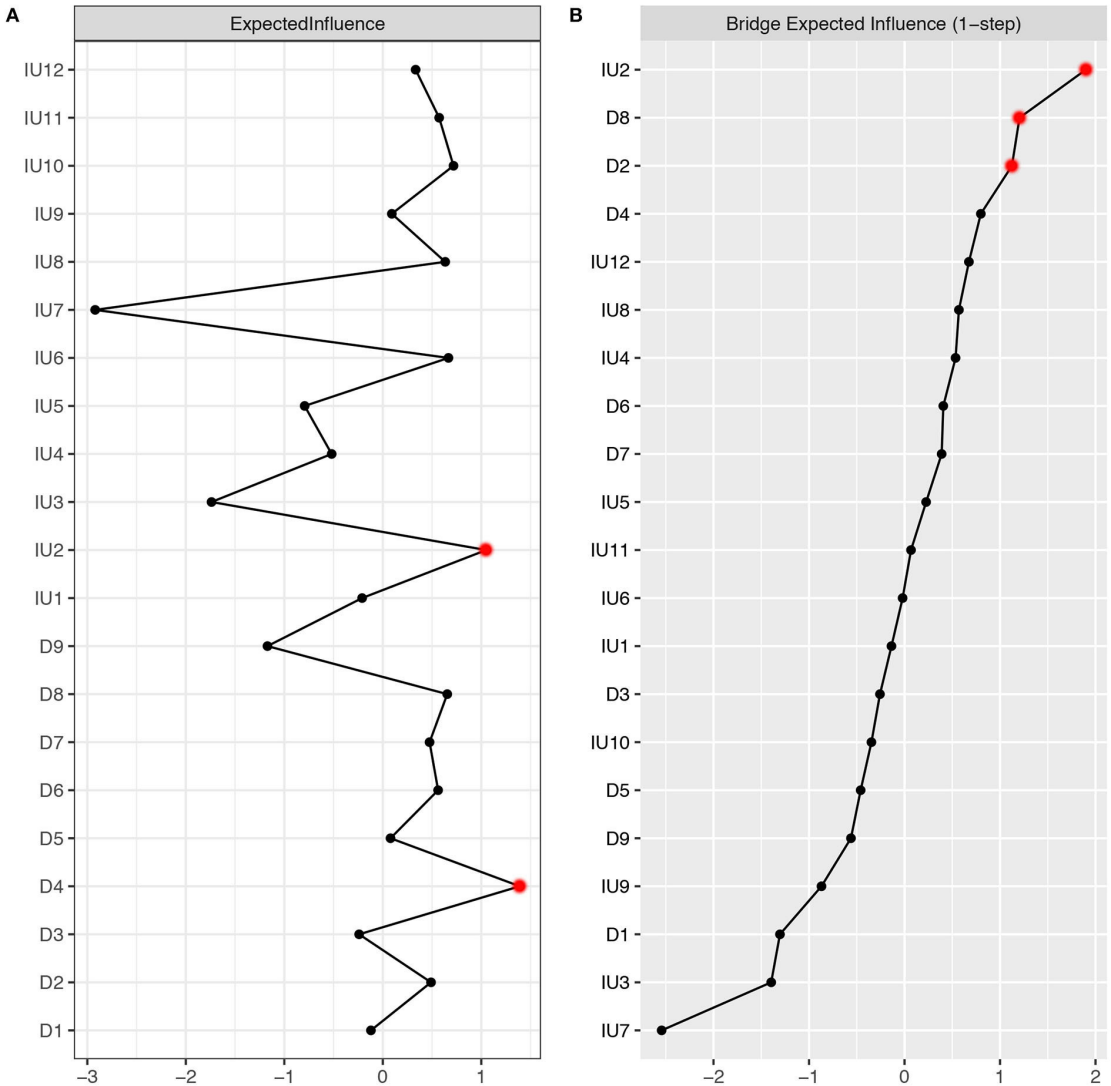
**(A)** Centrality plot depicted the expected influence (z-score) of each variable chosen in the final network. **(B)** Centrality plot depicted the bridge expected influence (z-score) of each variable chosen in the final network. The text of IU and depression can be seen in Table 1.

Figure 2A showed the node expected influence. Two variables with the highest expected influence were D4 “Fatigue” and IU2 “Frustration when facing uncertainty.” Thus, from the perspective of statistics, these two variables had the strongest associations with other variables in the present network. The CS-coefficient of node expected influence was 0.67 which indicates that the estimation of node expected influences was adequately stable (Supplementary Figure 3). Supplementary Figure 4 showed the bootstrapped difference test for node expected influences.

### Node bridge expected influence

Figure 2B showed the node bridge's expected influence. In the community of depression, two variables with the highest bridge expected influence were D8 “Psychomotor agitation/retardation” and D2 “Depressed or sad mood.” In the community of IU, one variable with the highest bridge expected influence was IU2 “Frustration when facing uncertainty.” Thus, from the perspective of statistics, IU2 had the strongest association with depression symptoms. The CS-coefficient of node bridge expected influence was 0.44 (>0.25), indicating that the node bridge expected influence calculation fulfilled the criteria (Supplementary Figure 5). The bootstrapped difference test for node bridge expected influences were shown in Supplementary Figure 6.

## Discussion

This was the first study to apply network analysis to examine the differential associations between symptoms of depression and components of IU during the COVID-19 pandemic. We found several pathways between IU and Depression, with the strongest emerging between IU2 “Frustration when facing uncertainty” and D6 “Feelings of worthlessness.” Our results also highlighted the important role of IU2, which was identified as both a central node and a bridge node within the estimated network. Other influential nodes were D4 “Fatigue” (with the highest node expected influence), D8 “Psychomotor agitation/retardation” (with high bridge expected influence), and D2 “Depressed or sad mood” (with high bridge expected influence).

In line with previous studies (24, 25, 43–46), the intra-community connections were generally denser and stronger than the inter-community connections within the estimated network. The strongest intra-community edge emerged between IU1 “Upset when facing unforeseen events” and IU2 “Frustration when facing uncertainty,” which was consistently reported in IU-related network analytic studies (24, 25). Within the depression community, we found that D3 “Sleep difficulties” and D4 “Fatigue” were closely related to each other, with “Sleep difficulties” may lead to “Fatigue” and vice versa. This finding is consistent with previous studies exploring the network structure of depression among college students (47, 48), the adult population (17, 49), domestic workers (50), and patients with epilepsy (51). These consistent findings addressed the concerns over the replicability of network analysis (52) and suggested that some specific symptoms pathway may exist across demographically different groups.

Regarding inter-community pathways, we found IU components may sustain distinct pathways leading to cognitive (e.g., D6 “Feeling of worthlessness”), emotional (e.g., D2 “Depressed or sad mood”), and somatic (e.g., D8 “Psychomotor agitation/retardation” and D4 “Fatigue”) symptoms of depression. The strongest pathway was observed between IU2 “Frustration when facing uncertainty” and D6 “Feelings of worthlessness.” This pathway may be particularly relevant when considering the cultural context. Specifically, emotional reactions to uncertainty (i.e., feeling frustrated) may be perceived as a lack of self-control or inability to restrain one's emotion, which is considered a major characteristic weakness of an individual and is against related social expectations (53–55). This may, in turn, promote self-hatred cognitions such as a “Feeling of worthlessness.” The finding supported the notion that cultural-specific factors should be considered when aiming to understand the maintenance of psychopathology. Specifically, it has been found that the “Feeling of worthlessness” is a uniquely important symptom among individuals from collectivistic cultural backgrounds (e.g., China and India) (56, 57), with unable to fulfill social expectations being proposed as a core mechanism underlying the maintenance of depression (56). Hence, it may be beneficial to replicate our findings among individuals from individualistic cultural backgrounds to ascertain whether cultural differences may impact the putative pathway between IU and depression.

Depression symptom D4 “Fatigue” and IU component IU2 “Frustration when facing uncertainty” showed the highest expected influence, indicating these two variables may be core to the maintenance of the IU-Depression network. The highest expected influence for IU2 is also consistent with our previous network study investigating IU-anxiety and IU-problematic smartphone use networks (24, 25). Depression symptom “Fatigue” is also a core symptom in previous network studies investigating the symptom network of depression-anxiety in college students and Filipino domestic workers (47, 48, 50).

When examining the bridge expected influence, we found that IU2 “Frustration when facing uncertainty,” D8 “Psychomotor agitation/retardation,” and D2 “Depressed or sad mood” emerged as bridging nodes between the IU and depression communities. As for IU, node bridge expected influence may reveal the unique effect of different components of IU on the various symptoms of depression. IU2 has the highest bridge expected influence. This indicates that IU2 has stronger connections with the depression community than other IU components. Therefore, from the perspective of the network system, targeting IU2 may be more effective at alleviating depression symptoms than targeting other components of IU. It should be noted that this is only a hypothesis, which needs to be tested experimentally and clinically. From the standpoint of concept, people are more likely to fear missing out and tend to get more information about the pandemic due to the uncertainty of the COVID-19 pandemic (58). However, it is almost impossible to obtain all the information about the COVID-9 pandemic. Under this condition, individuals may begin to generate negative emotions, which in turn increase the severity of depression symptoms. It is worth mentioning that IU7 “Organizing everything in advance” has four pathways linking to the depression community and three of them are negative. This leads to its lowest bridge centrality and may represent a protective ability for depression symptoms. In fact, organizing things in advance is a sign of maturity in Chinese culture, which might also symbolize the advantageous response as a substitute for intolerance when dealing with uncertainty (24). In the depression community, symptoms D8 and D2 have the greatest bridge expected influence. This implies that these two depression symptoms might be susceptible to the IU community.

We found that IU2 “Frustration when facing uncertainty” may act as both a central node and a bridge node within the IU-Depression network. This replicated previous findings from networks involving co-occurring anxiety (24) and problematic smartphone use (25). These consistent findings support the notion that IU may act as a transdiagnostic risk factor for various psychological conditions (e.g., emotional disorders, obsessive-compulsive disorder, addiction, and eating disorders) and add incremental value to current knowledge by teasing out the specific component that may underpin the association between IU and psychological conditions. This finding may have implications at the public health level. Specifically, by assessing individual differences in negative emotional reactions (i.e., frustrations) toward uncertainty, mental health providers may be able to identify the high-risk population for developing emotional and addiction-related symptoms. Further, interventions targeting this specific component may concurrently reduce various psychological conditions. This may be particularly relevant to reducing the public mental health burden during and after the pandemic.

Several limitations should be considered when interpreting current findings. First, the utilization of a student sample from a single Chinese university may limit the representativeness of current findings. Second, the current study used a cross-sectional approach. This means that no causal relationship can be established among study variables. Third, the network structure in the current study was examined at a group level and may not be replicable when examined at an individual level.

## Conclusion

Notwithstanding the limitations above, the current study has some strengths. To the satisfaction of our knowledge, our study is the first to apply network analysis to explore the component-to-symptom connections between IU and depression during the COVID-19 pandemic. Findings identify some central and bridge variables (especially IU component IU2 “Frustration when facing uncertainty”) in the depression-IU network. These central and bridge variables may also provide some insights for related preventions and therapies to address the COVID-19 pandemic's mental health needs. Based on our results, “Frustration when facing uncertainty” may be a promising target when designing interventions for depression symptoms.

## Data availability statement

The data analyzed in this study is subject to the following licenses/restrictions: In order to protect private information, the data cannot be made publicly available. The data may be available from the corresponding author for a reason, and requests to access these datasets should be directed to LR.

## Ethics statement

The studies involving human participants were reviewed and approved by the Declaration of Helsinki and was approved by the Ethics Committee of the First Affiliated Hospital of the Fourth Military Medical University (Project No.BWS16J012). The questionnaire was completed online in the WeChat application after electronic informed consent was obtained.

## Author contributions

TF, LR, HW, and XL developed the study idea and design. TF, LR, and CL wrote the original draft of this manuscript. All authors contributed to revising subsequent versions of the article.

## Funding

XL's involvement in this research was funded by the Fourth Military Medical University (BWS16J012). L-BC's involvement in this research was funded by the Fourth Military Medical University (2021JSTS30).

## Conflict of interest

The authors declare that the research was conducted in the absence of any commercial or financial relationships that could be construed as a potential conflict of interest.

## Publisher's note

All claims expressed in this article are solely those of the authors and do not necessarily represent those of their affiliated organizations, or those of the publisher, the editors and the reviewers. Any product that may be evaluated in this article, or claim that may be made by its manufacturer, is not guaranteed or endorsed by the publisher.
