# Supplementary material for: The relations between different components of intolerance of uncertainty and symptoms of depression during the COVID-19 pandemic: A network analysis
Source: Front Psychiatry. 2022 Oct 13;13:993814. doi: 10.3389/fpsyt.2022.993814 (PMC9613443; doi:10.3389/fpsyt.2022.993814)
Supplement: Supplementary file 2 [file Data_Sheet_2.docx]

**Supplementary Materials 1**

1. Figure S1. Accuracy of edge weights
2. Figure S2. Bootstrapped difference test for edge weights
3. Figure S3. Stability of node expected influences
4. Figure S4. Bootstrapped difference test for node expected influences
5. Figure S5. Stability of node bridge expected influences
6. Figure S6. Bootstrapped difference test for node bridge expected influences

Figure S1. Accuracy of edge weights

*Note*: The red line depicts the sample edge weights and the gray bar depicts the bootstrapped confidence interval.

Figure S2. Bootstrapped difference test for edge weights

*Note*: Gray boxes indicate edge weights that do not differ significantly from one another, while black boxes indicate edge weights that do differ significantly. Blue and red boxes on the diagonal correspond to edge weights with positive and negative correlations, respectively.

Figure S3. Stability of node expected influences

*Note*: The red bar represents the average correlation between node expected influences in the full sample and subsample with the red area depicting the 2.5th quantile to the 97.5th quantile.

Figure S4. Bootstrapped difference test for node expected influences

*Note*: Gray boxes indicate node expected influences that do not differ significantly from one another, while black boxes indicate node expected influences that do differ significantly. The number in the white boxes (i.e., diagonal line) represent the value of node expected influences.

Figure S5. Stability of node bridge expected influences

*Note*: The red bar represents the average correlation between node bridge expected influences in the full sample and subsample with the red area depicting the 2.5th quantile to the 97.5th quantile.

Figure S6. Bootstrapped difference test for node bridge expected influences

*Note*: Gray boxes indicate node bridge expected influences that do not differ significantly from one another, while black boxes indicate node bridge expected influences that do differ significantly.
